# Start-Up Characteristics of a Granule-Based Anammox UASB Reactor Seeded with Anaerobic Granular Sludge

**DOI:** 10.1155/2013/396487

**Published:** 2013-12-23

**Authors:** Lei Xiong, Yun-Yan Wang, Chong-Jian Tang, Li-Yuan Chai, Kang-Que Xu, Yu-Xia Song, Mohammad Ali, Ping Zheng

**Affiliations:** ^1^School of Metallurgy and Environment, Central South University, Changsha 410083, China; ^2^National Engineering Research Center for Control and Treatment of Heavy Metal Pollution, Changsha 410083, China; ^3^Department of Environmental Engineering, Zhejiang University, Zijingang Campus, Hangzhou 310058, China

## Abstract

The granulation of anammox sludge plays an important role in the high nitrogen removal performance of the anammox reactor. In this study, anaerobic granular sludge was selected as the seeding sludge to start up anammox reactor in order to directly obtain anammox granules. Results showed that the anammox UASB reactor was successfully started up by inoculating anaerobic granular sludge, with substrate capacity of 4435.2 mg/(L*·*d) and average ammonium and nitrite removal efficiency of 90.36% and 93.29%, respectively. During the start-up course, the granular sludge initially disintegrated and then reaggregated and turned red, suggesting the high anammox performance. Zn-Fe precipitation was observed on the surface of granules during the operation by SEM-EDS, which would impose inhibition to the anammox activity of the granules. Accordingly, it is suggested to relatively reduce the trace metals concentrations, of Fe and Zn in the conventional medium. The findings of this study are expected to be used for a shorter start-up and more stable operation of anammox system.

## 1. Introduction

Anaerobic ammonia oxidation (anammox) is one of the latest additions to the biogeochemical nitrogen cycle initially discovered in the 1990s [1]. It involves the autotrophic oxidation of ammonium to dinitrogen gas using nitrite as electron acceptor under anaerobic conditions (1) [1, 2]. The process is performed by microorganisms belonging to the order Brocadiales and affiliated to the Planctomycetes:

(1) NH4++1.32NO2−+0.066HCO3−+0.13H+→1.0N2+0.26NO3−+0.066CH2O0.5N0.15+2.03H2O

In contrast to conventional biological nitrogen removal processes, anammox presents advantages in less operational costs and higher nitrogen removal efficiency due to its low dependency of oxygen, none organic carbon consumption, and less sludge production [2, 3]. Thus, anammox has attracted much attention in the fields of environmental science and engineering. Several full-scale anammox plants have been employed for nitrogen removal from ammonium-rich wastewaters with maximum nitrogen removal rate (NRR) up to 9500 mg/(L · d) [3, 4].

However, the anammox microbes are characterized by a very slow growth rate with their doubling time best estimated at 7–11 d [5]. The enrichment of anammox bacteria from a mixed inoculum requires the optimization of conditions favorable for the anammox bacteria and generally takes 200–300 days [3, 6, 7]. The long start-up has been becoming one choke point on the application of the anammox process.

Study of anammox start-up has been focused on factors that have been found to have an impact on cultivation of anammox bacteria including hydraulic retention time (HRT), dissolved oxygen (DO), inoculum, temperature, wastewater composition, nitrogen compound concentration, and reactor configuration [8–15]. Among researches on anammox start-up course seeded by various conventional sludges [11, 16–23], anaerobic granular sludge has been reported as a preferable inoculum based on compact structure with very good settling properties, high removal efficiency, and strong shock load capacity [24]. During the start-up course, the anaerobic granular sludge can be directly converted into red anammox granules, which plays a crucial role in stable and efficient operation of anammox reactor. However, researches on start-up characteristics of anammox seeded with anaerobic granular sludge are still inadequate, and anaerobic granular sludge's variable properties are currently unclear. In this study, a granule-based anammox UASB reactor seeded with anaerobic granular sludge was started up, and start-up properties were investigated.

## 2. Materials and Methods

### 2.1. Anammox Reactor

**Figure 1:**
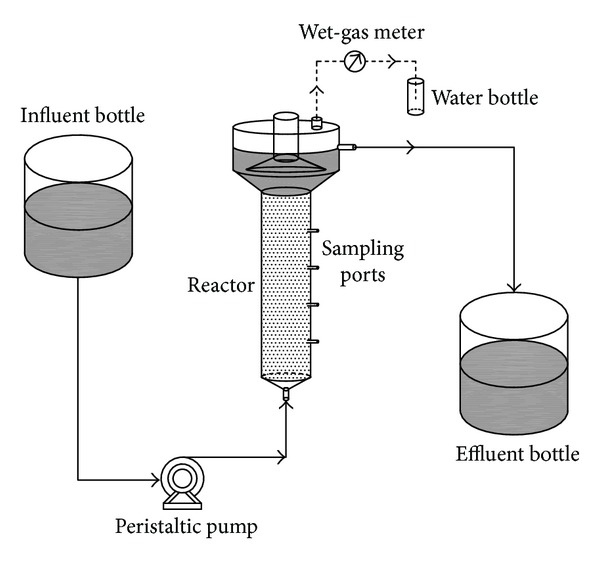
Schematic diagram of the anammox UASB system.

A plexiglass upflow anaerobic sludge blanket (UASB) reactor (Figure 1) was used in this study. The reactor had a functional working volume of 1.0 L with an internal diameter of 0.5 dm. It was covered with black cloth as conducted by van der Star et al. (2008) [5] to avoid light inhibition, growth of phototrophic organisms, and subsequent oxygen production. The reactor was operated at the temperature of 35 ± 1°C by using a thermostatic water bath.

### 2.2. Synthetic Wastewater

In this study, the anammox reactor was constantly fed with synthetic wastewater based on tap water, which contained NaH_2_PO_4_ 0.01 g/L, CaCl_2_ · 2H_2_0 0.0056 g/L, MgS0_4_ · 7H_2_0 0.3 g/L, KHCO_3_ 1.25 g/L and 1 ml/L trace element solution I and II. The concentration of nitrite (NaNO_2_) and ammonium ((NH_4_)_2_SO_4_) was supplied as needed. Trace element solution I consisted of FeSO_4_ 5 g/L, EDTA 5 g/L. Trace element solution II consisted of EDTA 15 g/L ZnSO_4_ · 7H_2_O 0.430 g/L, CuSO_4_ · 5H_2_O 0.250 g/L, NiCl_2_ · 6H_2_O 0.190 g/L, NaMoO_4_ · 2H_2_O 0.22 g/L, and H_3_BO_4_ 0.014 g/L [25]. The pH of the influent was set at 6.8.

### 2.3. Seed Sludge

The seeding anaerobic granular sludge was taken from an IC reactor in a paper mill wastewater treatment plant located in Hunan Province, China. The characteristics of the seeding sludge are illustrated in Table 1. Before the inoculation, seed granules were washed several times using phosphate buffer (0.14 g/L KH_2_PO_4_ and 0.75 g/L K_2_HPO_4_) until the supernatant COD concentration decreased to 10 mg/L.

### 2.4. Analytical Procedures

The influent and effluent samples were collected on daily basis and were analyzed immediately. Ammonium, nitrite, and nitrate concentrations were determined spectrophotometrically; biomass concentrations were determined as volatile suspended solids (VSS), according to standard methods (APHA, 1998 [26]). Cu and Fe concentrations in the wastewater samples were determined through ICP-AES (iCAP6300). The pH of the wastewater samples was determined by the HANNA pH 211 type acidometer with a selective electrode. Digital macrophotography was performed by the Canon EOS 600D Digital Single Lens Reflex. The energy dispersive spectrum (EDS) is acquired based on the SEM technique.

### 2.5. SEM

Morphological characteristics of the biomass specimens were observed using SEM model JEOL JSM-6360LV. The biomass specimens were fixed by glutaraldehyde in paraformaldehyde solution for 3 h, subsequently, dehydrated through a graded series of ethanol solution 25%, 50%, 75%, 90%, and 100% (thrice for each concentration), then gold-coated by a sputter, and finally observed under scanning electron microscopy.

## 3. Results and Discussion

### 3.1. Start-Up and Performance of the Anammox Reactor

The start-up of anammox reactor, essentially includes activation of bacteria in the system, biomass expansion, and the enhancement of reaction [12]. In this study, reducing hydraulic retention time (HRT) and increasing the inflow nitrogen concentration were alternatively adopted to increase volumetric nitrogen loading rate of the reactor.

**Figure 2:**
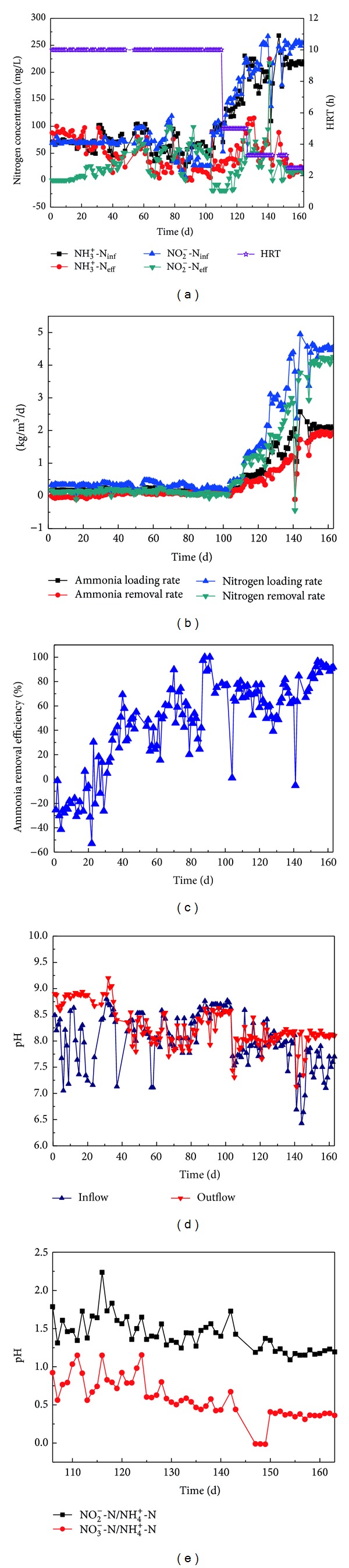
Performance of the anammox reactor.

According to Tang et al. (2009) [12], the overall start-up of the anammox reactors was divided into four phases, namely, cell lysis phase, lag phase, activity elevation phase, and stationary phase. During cell lysis phase (days 1–29), ammonium concentration of outflow exceeded that of inflow, and ammonium removal rate was below zero (Figure 2(a)). These phenomena could be attributed to the autolysis of heterotrophic bacteria, which were the dominant microbe of seed sludge. Heterotrophic bacteria went through starving and then died when exposed in medium absence of organic, releasing ammonium to the water through mineralization and finally leading to higher outflow ammonium concentration over inflow [17]. Disintegration of granular sludge was observed by visual observation in the reactor at this time. The autolysis of heterotrophic bacteria released organic matter simultaneously, which was used by denitrifying bacteria as electron donor to denitrify NO_2_^−^-N to nitrogen gas. The effluent nitrite concentration was detected to be zero; meanwhile, apparent gas production was observed in the reactor, suggesting that denitrification did occur in the reactor. In addition, the observed higher outflow pH (Figure 2(d)) further confirmed the hypothesis since denitrification is alkali-induced reaction. As shown in Figure 2(a), the effluent ammonium concentration at the beginning and the end of lag phase was 87.78 mg/L and 62.22 mg/L (when influent ammonium concentration was average 70 mg/L), with the corresponding ammonium removal rate of −42.67 mg/(L · d) and 31.11 mg/(L · d), respectively, (Figure 2(b)), indicating the attenuation of bacteria autolysis. Accordingly, the organic matter (electron donor) in the reactor decreased and denitrification reduced gradually, resulting in the increase of effluent nitrite concentration. The effluent nitrite concentration at the beginning and the end of lag phase was 0 and 7 mg/L (when influent nitrite concentration was average 70 mg/L), with nitrite removal rate slightly decreased from 165 mg/(L · d) to 145 mg/(L · d).

On day 30, the influent and effluent ammonium concentrations were nearly the same, indicating that the start-up turned into the lag phase [12]. This phase was characterized by sharp variations in effluent ammonium concentration (Figure 2(a)) and ammonium removal efficiency (Figure 2(c)). Yang et al. [14] seeded anaerobic granular sludge to start up anammox reactor, and the lag phase lasted for 27 days. Even under sulfate press and seed with nitrifying sludge, the time of the lag phase to start an anammox reactor was no more than 49 day. However, in the present study, no signal indicating the end of lag phase was observed after 73 days of lag phase.

As reported by van der Star et al. [3] and Tang et al. [10, 23, 27, 28], addition of a very small amount of red anammox sludge could significantly enhance the anammox performance in the reactor system, which was confirmed by the experience of start-up of the first full scale anammox reactor in Rotterdam, The Netherland [3]. Therefore, in order to shorten the lag phase and accelerate start-up of the anammox UASB reactor, 10 mL matured red anammox sludge cultivated in our lab-scale high-rate anammox reactors was added to the tested reactor. Soon after the addition, anammox reaction appeared and the nitrogen removal efficiency enhanced continuously, suggesting that the start-up course turned into activity elevation phase (104–148 d). During this phase, ammonium and nitrite concentrations of influent were progressively increased from both 70 mg/L to 229 mg/L and 252 mg/L, respectively. Meanwhile, HRT was reduced from 10 h to 2.5 h gradually. The nitrogen loading rate was continuously elevated from 336 mg/(L · d) to 4617.6 mg/(L · d), along with a progressive increase in the ammonium removal rate from 95.47 mg/(L · d) to 1722 mg/(L · d), and the total nitrogen removal rate was significantly increased to 3768 mg/(L · d). At the same time, effluent nitrite concentration was maintained below 50 mg/L during the whole operation. Based on the mass balance, the percentage of nitrite conversion via anammox rose from 25% to 96%, which indicated that anammox finally dominated the system. Effluent nitrate concentration increased with the average value of 68.73 mg/L. The stoichiometric ratio of the ammonium conversion, nitrite removal, and nitrate production was 1 : 1.26 : 0.44 (Figure 2(e)), which was close to the theoretical value (1 : 1.32 : 0.26) reported by Strous et al. [2]. Higher nitrate production in this research might be associated with trace oxide brought by tap water due to the predeoxidized treatment with influent. During this period, granules' color turned from black to brown and then red subsequently, implying the significant enhancement in the activity of anammox bacteria.

Based on the start-up standard proposed by Jin et al. (2008) [29] (the nitrogen removal rate was higher than 0.5 kg/(m^3^ · d)), the anammox UASB reactor was successfully started up. However, the subsequent increase of nitrite concentration after reaching 252 mg/L no longer improved the volumetric substrate nitrogen removal rate (149–164 d), the function of anammox became close to saturation, and the start-up course came into the stationary phase [12]. In order to further increase ammonium removal efficiency and consequently lower effluent ammonium concentration, the ratio of ammonium and nitrite concentration in the influent was set at 1 : 1.2, on the basis of practical reacting ratio value. The reactor ran steadily, certified by ammonium and nitrite removal efficiency up to 95.35% and 100%. Under the total nitrogen loading rate of 4435.2 mg/(L · d), the ammonium and nitrite concentrations in effluent were below 50 mg/L and 25 mg/L, respectively, attaining average ammonium and nitrite removal rate of 1841.6 and 2209.9 mg/(L · d), respectively.

### 3.2. Characteristics of the Anammox Granules during the Start-up

**Figure 3:**
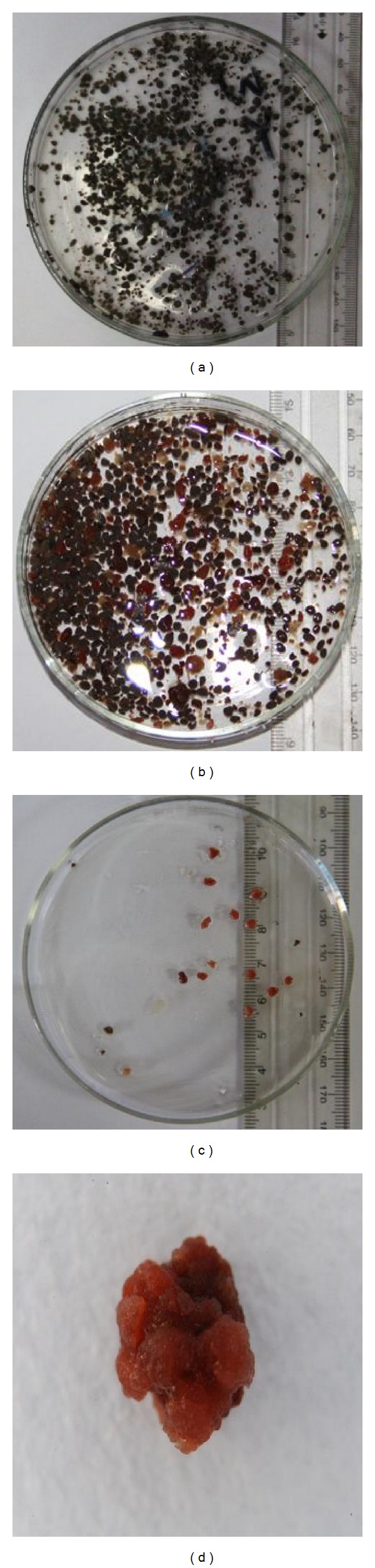
Photograph of granules of biomass (a, b, and c) and a granule (d) on day 0 (a) and day 150 (b, c, and d).

**Figure 4:**
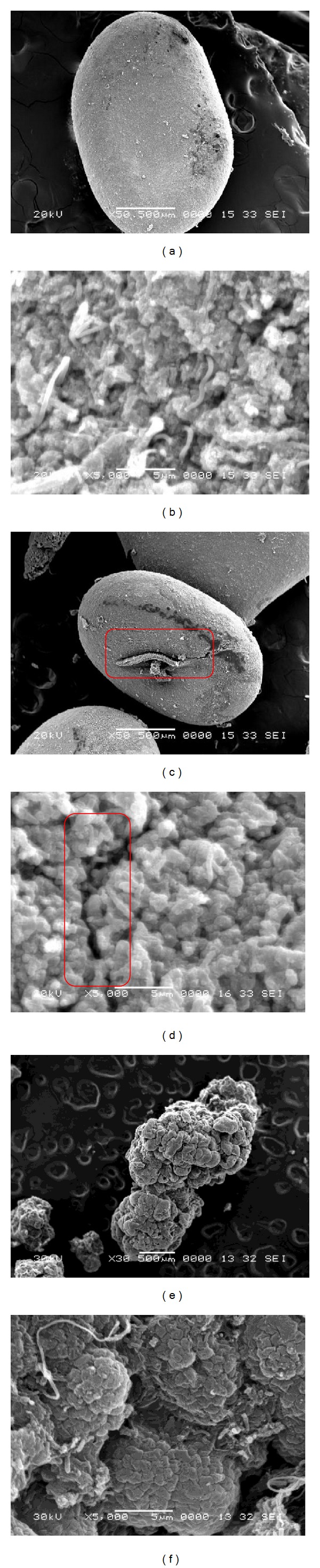
SEM observation of the sludge on day 0 (a, b), day 45 (c, d), and day 150 (e, f) of the start-up. The red square indicates the crack on the granular surface.

The apparent characteristics of the seeding anaerobic granular sludge taken from an IC reactor treating paper mill wastewater are shown in Figure 3(a). As shown in Figure 3(a), the anaerobic granules possessed black color and close-to-sphere shape, with a diameter range of 1–3 mm. Scanning electron microscopy (SEM) was used to observe the surface of aggregates along the experiment. The surface of the anaerobic granules was smooth without any acute angle and mainly consisted of bacilli. Filamentous bacteria were also observed (Figures 4(a) and 4(b)).

On day 45, in the start-up course, distinct crack appeared along with the generation of sediment, which has not been mentioned in the anammox literature seen (Figure 4(c)). Moreover, the granular surface became rough. These may be associated with the autolysis of bacteria in the reactor, as illustrated above. Since there is no continuous supply of organic matter, the aboriginal heterotrophic bacteria in the anaerobic granular sludge were starved to death, which might be a leading reason for the granular disintegration.

On day 140, the volumetric nitrogen removal rate was up to 3000 mg/(L · d) after the anammox reactor was started up. According to Tang et al. [10], Schmid et al. [30], and Klotz et al. [31], anammox bacteria contained two key enzymes rich in heme *c*, that is, hydroxylamine oxidoreductase and hydrazine oxidoreductase, both of which are important during the anammox metabolic pathway. Thus, anammox granule possessed uniquely carmine color. As shown in Figure 3(b), the proportion of carmine anammox granules was notably larger at this time. The new generated anammox granules were inordinately shaped in contrast with the anoxic seeding granules, with a diameter range of 2-3 mm (Figure 3(d)). Furthermore, SEM observations showed the granules with a high degree of compactness, compact areas on the surface of the granules, fissures with irregular depression surface, morphology, and the sludge hollow phenomenon (Figures 4(e) and 4(f)).

### 3.3. Precipitation on the Granular Surface

**Figure 5:**
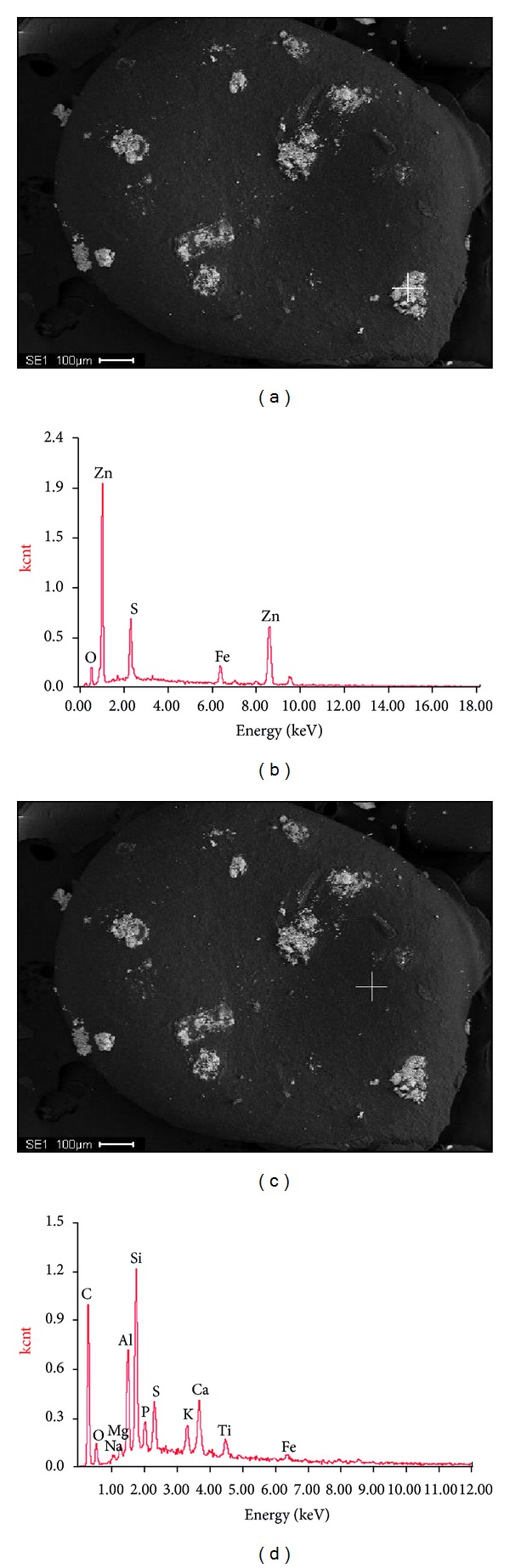
Elemental analysis based on SEM-EDS carried out on day 45 of the start-up, indicating the percentage composition by the mass of the most abundant elements of the precipitate (a, b) and other areas on biomass surface (c, d), respectively.

When granular sludge was observed by SEM with a broad view at low magnification, sediments adhering to the granular surface were discovered, as indicated in Figures 5(a) and 5(c). In order to exactly analyze the mineral contents of the precipitation, EDS analysis based on SEM was carried out in the present study. Results showed that the main elements in the sediment were O, S, Fe, and Zn, among which Zn and Fe took a more ascendant part with mass percent of 72% and 8.47% and atomic number ratio of 53% and 7.26%, respectively. It showed that a series of precipitation reactions may occur to generate insoluble Fe-Zn deposits in the reactor. This is a new discovery that was not available in references and would be a new field for the subsequent anammox research.

Based on ICP-AES analysis, the trace metals Fe, and Zn concentrations in the influent and effluent were 1.3, 0.8, and 1.0, 0.3 mg/L, respectively. Based on the mass balances, 0.3 and 0.5 mg/L decrements of Fe and Zn concentrations were observed. Fe and Zn were relatively excess in effluent, revealing that trace elements Fe and Zn were considerably surplus for anammox start-up. Since the number of anammox bacteria was very low in the initial start-up phases, the removal of Fe and Zn by anammox metabolism might be very little. The observed Fe and Zn precipitate on granular surface further reduced their bioavailability in wastewater, which was not helpful for the anammox process especially in the start-up.

As reported by Trigo et al. (2006) [15], after feeding with the mineral medium proposed by van de Graaf et al. (1995) [25] with 226 mg/L CaCl_2_ · 2H_2_O and 50 mg/L KH_2_PO_4_ for 10 days, the reactor performance underwent a sharp decrease from 100 mg/(L · d) to 10 mg/(L · d). The red granules' color gradually changed to light chestnut with time that occurred simultaneously with the observed NVSS accumulation. Salt precipitation was observed and considered as a feasible reason of these phenomena. Elemental analysis of the biomass surface showed that mass percentage of 17.3% calcium and 7.8% phosphorus was detected, and the molar relationship between Ca and P was 1.71, which is close to that of 1.5 of calcium phosphate, indicating that the precipitates were formed by calcium salts, especially by the calcium phosphate salt. After decreasing the calcium and phosphorus concentrations to 5.65 mg/L CaCl_2_ · 2H_2_O and 10 mg/L KH_2_PO_4_, both the activity and the nitrogen uptake of the system increased quickly. A maximum nitrogen removal rate of 710 mg/(L · d) was obtained after 185 days' operation. The concentration of NVSS was kept approximately constant around 0.2 g/L, indicating that no additional salt precipitation took place. Additionally, biomass color varied from light chestnut to red color. Therefore, it is concluded that salts precipitation on the biomass surface interfered with microbial activity and caused a decrease of the nitrogen removal rate.

In this study, the optimized mineral medium of Trigo et al. (2006) [15] was used to cultivate anammox biomass. During the operation, no calcium phosphate precipitation was detected although the low distribution of calcium and phosphorus on the granular surface was observed (Figures 5(c) and 5(d)). However, Fe-Zn precipitation was surprisingly observed on the granular surface. The chemical precipitates might also interfere with microbial activity and, thus, delay the start-up of anammox reactor, as demonstrated above. Therefore, it is suggested by the present study to relatively reduce the Fe and Zn concentrations in the mineral medium to prevent Fe-Zn precipitation, under which microbial growth might be further ensured.

## 4. Conclusions

Anammox UASB reactor was successfully started up by inoculating anaerobic granular sludge in the present study. The nitrogen removal performance was enhanced to 4435.2 mg/(L · d) and HRT was minimized to 2.5 h, attaining average ammonium and nitrite removal efficiency of 90.36% and 93.29%, respectively. During the start-up course, the granular sludge experienced a process of initial disintegration and subsequent reaggregation. Disintegration of granules was probably caused by bacteria autolysis. In addition, Zn-Fe precipitation was firstly discovered on the surface of granules during the operation, which could impose inhibition to microbial activity and penalize the start-up of anammox reactor. Therefore, it is suggested to adequately decrease the concentrations of Fe and Zn in mineral medium to avoid Fe-Zn precipitation during the start-up of anammox reactors.

## Figures and Tables

**Table 1:** Physical and chemical characteristics of seeding sludge.

Seed sludge	SS/g·L^−1^	VSS/g·L^−1^	VSS/SS	Diameter/mm
Anaerobic granular sludge	50.6	42.5	0.84	1-2

## References

[1] Mulder A, Graaf AA, Robertson LA, Kuenen JG (1995). Anaerobic ammonium oxidation discovered in a denitrifying fluidized bed reactor. *FEMS Microbiology Ecology*.

[2] Strous M, Kuenen JG, Jetten MSM (1999). Key physiology of anaerobic ammonium oxidation. *Applied and Environmental Microbiology*.

[3] van der Star WRL, Abma WR, Blommers D (2007). Startup of reactors for anoxic ammonium oxidation: experiences from the first full-scale anammox reactor in Rotterdam. *Water Research*.

[4] Joss A, Salzgeber D, Eugster J (2009). Full-scale nitrogen removal from digester liquid with partial nitritation and anammox in one SBR. *Environmental Science and Technology*.

[5] van der Star WR, Miclea AI, van Dongen UG, Muyzer G, Picioreanu C, van Loosdrecht M (2008). The membrane bioreactor: a novel tool to grow anammox bacteria as free cells. *Biotechnology and Bioengineering*.

[6] Dapena-Mora A, Campos JL, Mosquera-Corral A, Jetten MSM, Méndez R (2004). Stability of the ANAMMOX process in a gas-lift reactor and a SBR. *Journal of Biotechnology*.

[7] Jetten M, Cirpus I, Kartal B (2005). 1994–2004: 10 Years of research on the anaerobic oxidation of ammonium. *Biochemical Society Transactions*.

[8] Suneethi S, Joseph K (2011). ANAMMOX process start up and stabilization with an anaerobic seed in anaerobic membrane bioreactor (AnMBR). *Bioresource Technology*.

[9] Tao Y, Gao D, Fu Y, Wu W, Ren N (2012). Impact of reactor configuration on anammox process start-up: MBR versus SBR. *Bioresource Technology*.

[10] Tang C-J, Zheng P, Hu B-L, Chen J-W, Wang C-H (2010). Influence of substrates on nitrogen removal performance and microbiology of anaerobic ammonium oxidation by operating two UASB reactors fed with different substrate levels. *Journal of Hazardous Materials*.

[11] Tsushima I, Kindaichi T, Okabe S (2007). Quantification of anaerobic ammonium-oxidizing bacteria in enrichment cultures by real-time PCR. *Water Research*.

[12] Tang C-J, Zheng P, Mahmood Q, Chen J-W (2009). Start-up and inhibition analysis of the Anammox process seeded with anaerobic granular sludge. *Journal of Industrial Microbiology and Biotechnology*.

[13] Bagchi S, Biswas R, Nandy T (2010). Start-up and stabilization of an Anammox process from a non-acclimatized sludge in CSTR. *Journal of Industrial Microbiology and Biotechnology*.

[14] Yang Z, Zhou S, Sun Y (2009). Start-up of simultaneous removal of ammonium and sulfate from an anaerobic ammonium oxidation (anammox) process in an anaerobic up-flow bioreactor. *Journal of Hazardous Materials*.

[15] Trigo C, Campos JL, Garrido JM, Méndez R (2006). Start-up of the Anammox process in a membrane bioreactor. *Journal of Biotechnology*.

[16] Pynaert K, Smets BF, Beheydt D, Verstraete W (2004). Start-up of autotrophic nitrogen removal reactors via sequential biocatalyst addition. *Environmental Science and Technology*.

[17] Chamchoi N, Nitisoravut S (2007). Anammox enrichment from different conventional sludges. *Chemosphere*.

[18] Nakajima J, Sakka M, Kimura T, Furukawa K, Sakka K (2008). Enrichment of anammox bacteria from marine environment for the construction of a bioremediation reactor. *Applied Microbiology and Biotechnology*.

[19] Quan Z-X, Rhee S-K, Zuo J-E (2008). Diversity of ammonium-oxidizing bacteria in a granular sludge anaerobic ammonium-oxidizing (anammox) reactor. *Environmental Microbiology*.

[20] van de Vossenberg J, Rattray JE, Geerts W (2008). Enrichment and characterization of marine anammox bacteria associated with global nitrogen gas production. *Environmental Microbiology*.

[21] Ni B, Chen Y, Liu S, Fang F, Xie W, Yu H (2009). Modeling a granule-based anaerobic ammonium oxidizing (ANAMMOX) process. *Biotechnology and Bioengineering*.

[22] Lawrence KW, Howard HL, Yung TH (2005). *Waste Treatment in the Process Industries*.

[23] Tang C-J, Zheng P, Wang C-H, Mahmood Q (2010). Suppression of anaerobic ammonium oxidizers under high organic content in high-rate Anammox UASB reactor. *Bioresource Technology*.

[24] Franco A, Roca E, Lema JM (2006). Granulation in high-load denitrifying upflow sludge bed (USB) pulsed reactors. *Water Research*.

[25] van de Graaf AA, Mulder A, De Bruijn P, Jetten MSM, Robertson LA, Kuenen JG (1995). Anaerobic oxidation of ammonium is a biologically mediated process. *Applied and Environmental Microbiology*.

[26] American Public Health Association (APHA) (1998). *Standard Methods for the Examination of Water and Waste Water*.

[27] Tang C-J, Zheng P, Wang C-H (2011). Performance of high-loaded ANAMMOX UASB reactors containing granular sludge. *Water Research*.

[28] Tang C-J, Zheng P, Zhang L (2010). Enrichment features of anammox consortia from methanogenic granules loaded with high organic and methanol contents. *Chemosphere*.

[29] Jin R-C, Zheng P, Hu A-H, Mahmood Q, Hu B-L, Jilani G (2008). Performance comparison of two anammox reactors: SBR and UBF. *Chemical Engineering Journal*.

[30] Schmid MC, Hooper AB, Klotz MG (2008). Environmental detection of octahaem cytochrome c hydroxylamine/hydrazine oxidoreductase genes of aerobic and anaerobic ammonium-oxidizing bacteria. *Environmental Microbiology*.

[31] Klotz MG, Schmid MC, Strous M, Op Den Camp HJM, Jetten MSM, Hooper AB (2008). Evolution of an octahaem cytochrome c protein family that is key to aerobic and anaerobic ammonia oxidation by bacteria. *Environmental Microbiology*.

