# Corrigendum to “Start-Up Characteristics of a Granule-Based Anammox UASB Reactor Seeded with Anaerobic Granular Sludge”

**DOI:** 10.1155/2017/4629516

**Published:** 2017-11-07

**Authors:** Lei Xiong, Yun-Yan Wang, Chong-Jian Tang, Li-Yuan Chai, Kang-Que Xu, Yu-Xia Song, Mohammad Ali, Ping Zheng

**Affiliations:** ^1^School of Metallurgy and Environment, Central South University, Changsha 410083, China; ^2^National Engineering Research Center for Control and Treatment of Heavy Metal Pollution, Changsha 410083, China; ^3^Department of Environmental Engineering, Zhejiang University, Zijingang Campus, Hangzhou 310058, China

In the article titled “Start-Up Characteristics of a Granule-Based Anammox UASB Reactor Seeded with Anaerobic Granular Sludge” [1], there were two errors in Figure 2, where NH_3_^+^ in the legend of Figure 2(a) should be changed to NH_4_^+^ and the *x*-axis in Figure 2(e) should be changed from “pH” to “Ratio.” The corrected figure is as follows.

## Figures and Tables

**Figure 2:**
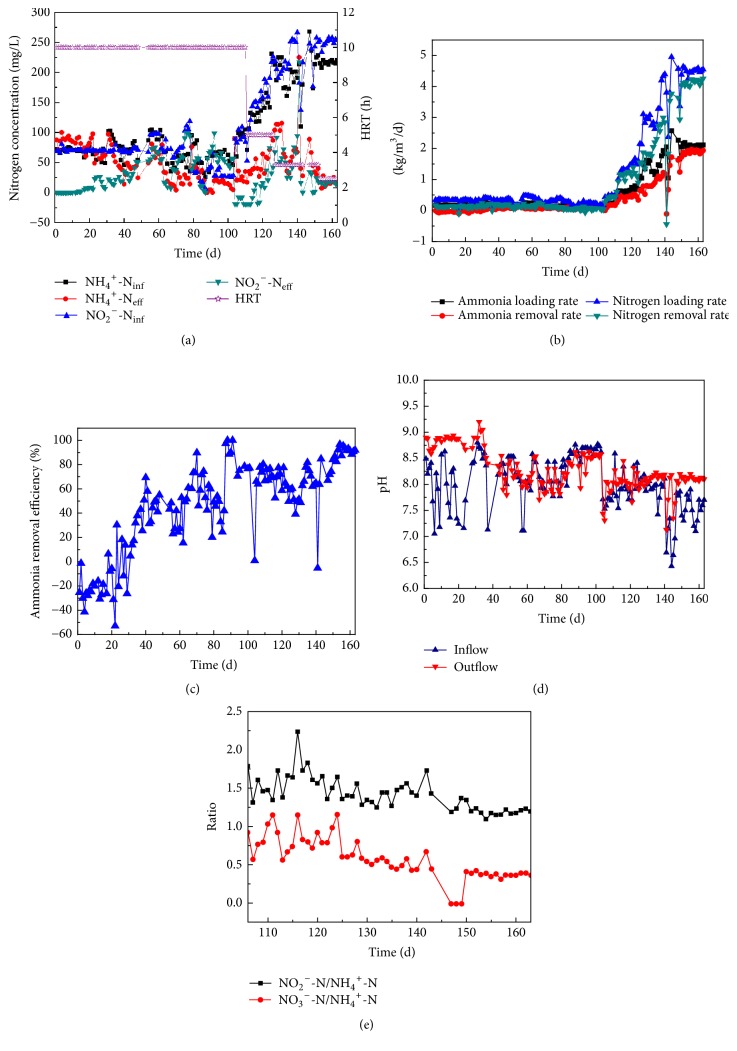
Performance of the anammox reactor.
